# A Comparison of the Efficacy of Immediate *Versus* Delayed Frozen–Thawed Embryo Transfer on the Ongoing Pregnancy Rate After a Failed IVF Attempt: Study Protocol for a Randomized, Non-Inferiority, Parallel-Group, Controlled Trial

**DOI:** 10.3389/fendo.2021.603158

**Published:** 2021-02-18

**Authors:** Zhuo Liu, Fengyi Dong, Yunhan Wang, Mingming Zheng, Mengyang Song, Yixuan Wang, Jingyan Song

**Affiliations:** ^1^ Reproductive and Genetic Center of Integrated Traditional and Western Medicine, The Affiliated Hospital of Shandong University of Traditional Chinese Medicine, Jinan, China; ^2^ Child Rehabilitation Center, Jinan Maternity and Child Care Hospital, Jinan, China; ^3^ The First Clinical College, Shandong University of Traditional Chinese Medicine, Jinan, China

**Keywords:** randomized controlled trial, pregnancy, *in-vitro* fertilization, time interval, frozen–thawed embryo transfer

## Abstract

**Introduction:**

In clinical practice, the ideal time at which to perform a Frozen–thawed Embryo Transfer (FET) after a failed *In-vitro* Fertilization-embryo Transfer (IVF-ET) is still unclear to most practicing physicians. In addition, physicians often delay the introduction of FET due to concerns on the possible residual effects of ovarian hyperstimulation, which may interfere with the regular menstrual cycle. Moreover, given that most of the published studies on the topic are retrospective with contradictory findings, it is crucial to provide evidence-based randomized control guides for clinical practice.

**Methods/analysis:**

The study is a randomized, non-inferiority, parallel-group, controlled trial that will enroll a total of 732 women undergoing their first FET after a failed fresh embryo transfer (ET) cycle. The participants will then be randomized into two groups based on a computer-generated randomized list. The two groups include: (i) an immediate group were FET will be carried out during the first menstrual cycle after a failed fresh ET cycle and (ii) a delayed group where FET will be carried out during the second menstrual cycle after a failed fresh ET cycle. Primary outcomes will be defined as viable pregnancies with fetal heartbeats, diagnosed through pelvic ultrasonography after twelve weeks of gestation.

**Ethics and dissemination:**

The study was approved by the Ethics Committee of the Assisted Reproductive Medicine at the Affiliated Hospital of Shandong University of Traditional Chinese Medicine (SDTCM/E-2020.2.01). In addition, written informed consent will be obtained from all the participants before the study. The results of this trial will be disseminated in a peer-reviewed journal.

**Discussion:**

Currently, there is no consensus with regard to the duration after which the effects of ovarian stimulation are observed after a failed fresh ET and the optimal time required to begin FET. Moreover, no randomized controlled trial exists that compares the ongoing pregnancy rates after immediate versus delayed FET following a failed fresh ET cycle. Therefore, it is important to conduct a well-designed randomized trial to determine whether it is necessary to delay FET for at least one menstrual cycle after the failure of fresh ET.

**Clinical Trial Registration:**

ChiCTR2000033313 (http://www.chictr.org.cn/enIndex.aspx).

## Highlights

The present study is the first randomized controlled trial to attempt to accumulate significant clinical evidence and draw conclusions with regard to the ideal time to conduct FET after a failed fresh embryo transfer cycle.
The current protocol includes participants aged 21–44 years, undergoing their first FET after the fixed GnRH antagonist protocol with standard Controlled Ovarian Stimulation (COS) in the IVF/ICSI procedure. The results obtained will therefore be able to reflect the situation in a majority of women in the infertile population.
However, it will be impossible to blind the allocation of intervention measures to the participants, physicians, and researchers.
The sample size calculation in the current study may also not be able to detect smaller differences in the ongoing pregnancy rates of 10% between the immediate and delayed groups.

## Introduction

Since the early success achieved with FET three decades ago, embryo deferrals and cryopreservation have become an increasingly essential aspect of IVF treatment (1). In addition, women whose attempt at pregnancy failed after a fresh embryo transfer (ET) during a stimulated IVF cycle, often opt to immediately proceed with FET in order to get pregnant as soon as possible. However, the difference between fresh or frozen embryo transfers with regard to perinatal outcomes has been a subject of controversy. Notably, FET cycles have previously been associated with lower rates of *antepartum* hemorrhage (2), pre-term birth, ectopic pregnancy (3–6) and low birth weight (7–10). Nonetheless, FETs have been linked to higher rates of placental/hypertensive complications (5), large-for-gestational-age infants (5, 11) and inconclusive perinatal mortality rates (2, 11). Therefore, given the setbacks, most researchers are currently skeptical on the overall benefits of FET (12–15). In addition, physicians are generally questioned on ovarian hyperstimulation and the long-term effects on subsequent treatment (16). As such, FETs are often postponed in an attempt to minimize the possible residual effects of ovarian hyperstimulation on endometrial receptivity (17). However, existing literature on the area is largely scarce (18, 19) although deferrals by physicians even with the best of intentions, might frustrate couples.

Presently, limited evidence exists on the “perfect” timing for FET following a failed-fresh stimulated IVF cycle. Notably, two options can be considered following a failed-fresh cycle: the first one would be to perform FET during the first cycle (an immediate embryo transfer) while the other option would be to postpone for least one menstrual cycle (a delayed embryo transfer). In addition, two studies on failed fresh IVF showed no differences in the live birth rates or clinical pregnancies between delayed and immediate FET (20, 21). In contrast, Mass K et al. suggested that patients should not postpone FET after fresh ET failure. Additionally, Volodarsky-Perel et al. reported that FET should be postponed for at least one menstrual cycle, after a failed-fresh cycle (17, 22). Therefore, given the contradictory reports, it is necessary to provide evidence-based randomized control guides for clinical practice. Consequently, the present randomized clinical trial aims to compare the ongoing pregnancy rate after immediate *versus* delayed FET following a failed-fresh cycle. It is hypothesized that the ongoing pregnancy rates of the two options will be comparable. The following article is presented in accordance to the SPIRIT reporting checklist.

## Methods and Materials

### Study Design

**Figure 1:**
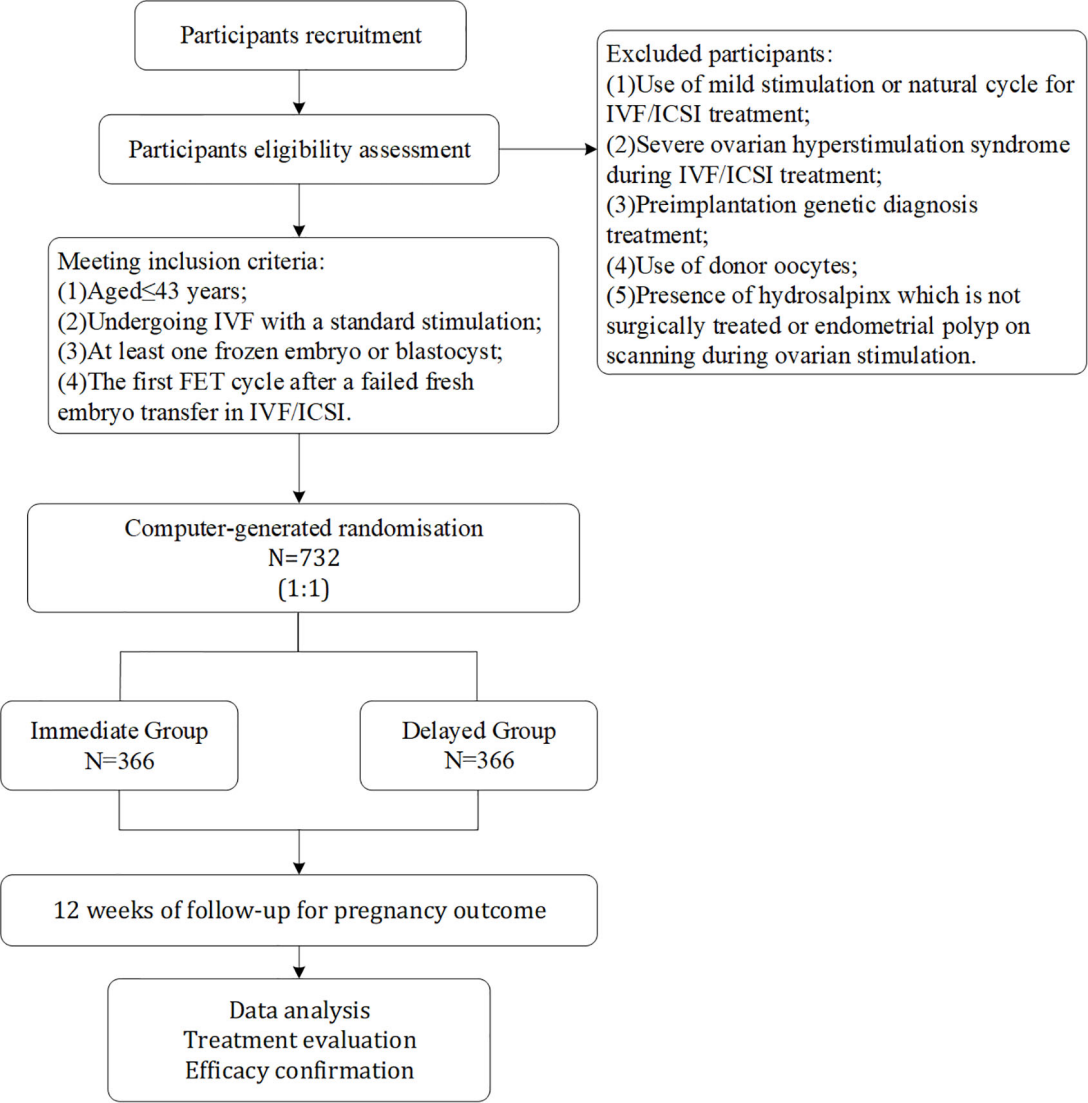
The study flowchart.

The study is a controlled single-center, randomized, parallel group, non-inferiority clinical trial conducted at the Affiliated Hospital of Shandong University of Traditional Chinese Medicine (TCM). An overview of the study visits is shown in **Table 1**, and the study flow chart is highlighted in **Figure 1**.

**Table 1:** Schedule of the study process (SPIRIT Figure).

	Study Period				
	Enrollment	Allocation	Post allocation		
Time point*	1st visit	2nd visit	3rd visit	Pregnancy visit	Follow-up visit
Preconception counselling	**√**	**√**	**√**	**√**	**√**
Eligibility screen	**√**				
Informed consent	**√**				
Questionnaire	**√**	**√**			
Laboratory index measurement (oocyte, zygote, embryo, *etc.*)	**√**				
Serological test for pregnancy		**√**	**√**	**√**	
Allocation (immediate FET or delayed FET)		**√**	**√**		
Menstrual cycle records			**√**		
Transvaginal ultrasound for pregnancy outcomes			**√**	**√**	**√**
Perinatal and neonatal outcomes records				**√**	**√**

### Participants

The participants will consist of women (who met the inclusion criteria) and their partners receiving IVF or ICSIs, recruited by physicians in the fertility clinics. Given that the number of patients who make daily visits is sufficient, there will be no need for additional strategies to ensure adequate recruitment. Thereafter, the participants will undergo detailed counseling and explanation from the doctors after which they will be required to sign the informed consent forms. Following this, eligible participants will be randomly allocated to either a delayed or an immediate group.

### Inclusion Criteria

Participants must be <44 years of age at the time of IVF/ICSI treatment.
Participants should have undergone an initial FET cycle after a failed fresh ET in IVF/ICSI.
Participants should be undergoing IVF/ICSI with a standard ovarian stimulation protocol.
Participants should have at least one frozen embryo remaining for transfer.

### Exclusion Criteria

Individuals with a Body Mass Index (BMI) ≥28 kg/m^2^.
Those using the natural cycle or mild stimulation for IVF/ICSI treatment.
Individuals with severe hyperstimulation ovarian syndrome during controlled ovarian stimulation.
Those with a history of unilateral oophorectomy or recurrent pregnancy loss, defined as two or more spontaneous abortions.
Acceptors of donated oocytes or performed either *In vitro* Maturation (IVM) or blastocyst biopsy for Preimplantation Genetic Diagnosis (PGD) or Preimplantation Genetic Testing for Aneuploidies (PGT-A).
Those previously diagnosed with congenital (*e.g*. mediastinal uterus and double uterus) or acquired (*e.g.* submucosal myoma and adenomyosis) uterine abnormalities.
Presence of a non-surgically treated hydrosalpinx or endometrial polyp and an ovarian endometriosis cyst requiring surgery, during ovarian stimulation.

Standard and mild stimulation in the study will be defined according to published ovarian stimulation terminologies for IVF (23). On the other hand, endometriosis will be diagnosed and classified according to guidelines by the European Society of Human Reproduction and Embryology (ESHRE) (24). Additionally, the Ovarian Hyperstimulation Syndrome (OHSS) will be classified and diagnosed according to guidelines by the Royal College of Obstetricians and Gynecologists (25).

### Randomization

Women undergoing the first FET cycle after a failed-fresh cycle will be randomized into two groups (immediate and delayed) according to a computer-generated randomization list. Randomization will be conducted on the day of the negative blood *β*-hCG test (fourteen days after fresh embryo transfer), for participants with a failed-fresh ET. In addition, randomization will be conducted by the study nurse blinded from the entire recruitment and clinical management of the participants through random number generation and grouping using the SPSS software (Version 26.0, IBM Corp., Armonk). Moreover, the same project nurse will prepare the randomization arm by placing them into opaque envelopes for subsequent use. Finally, participants will be randomized according to the opaque envelopes into one of two groups: 1) The immediate group in which FET will be performed after the first menstrual cycle following the failed-fresh cycle; 2) The delayed group in which FET will be performed after two or more menstrual cycles following the failed-fresh cycle.

### Blinding

Due to the nature of the study, it will be impossible to blind the participants and researchers. However, the embryologist, carrying out the assessments (embryo quality test) will be blinded to the intervention.

### Interventions

Participants will undergo the IVF/ICSI treatment as clinically indicated. In addition, fixed GnRH antagonist (GnRH-ant) (cetrorelix; Merck Serono, Darmstadt, Germany) protocol will be employed with 150–450 IU/day of recombinant FSH (Puregon, MSD, Courbevoie, France; Gonal-F, Merck-Serono, Lyon, France). Additionally, the doses of gonadotropin will be determined based on the characteristics of individual patients. Thereafter, oocyte retrieval will be conducted under ultrasound transvaginal guidance, 34–36 h after triggering with GnRH-a (Triptoreline, Decapeptyl, Ipsen, France) or recombinant hCG (rhCG, Ovitrelle, Serono, France). Additionally, the BD Falcon IVF medium (Becton, Dickinson and Company, Franklin Lakes, NJ, USA) will be used to collect the oocytes and perform embryo culture at 6% CO_2_, 5% O_2_ and 37.0°C (C200 CO_2_ Incubator, Labotect Labor-Technik-Göttingen GmbH, Göttingen, Germany). After this, oocytes will be fertilized based on the semen quality of individual participants’ partner, using either conventional insemination or ICSI following the standard protocol. Normal fertilization will then be assessed (a second polar body and two pronuclei) after 16–18 h of insemination or ICSI. Notably, an embryo with at least seven blastomeres (grades one and two) on the third day after oocyte retrieval will be defined as good quality. In addition, embryos with fragments <50% will be frozen. According to the standard protocol, all “good” embryos will be vitrified using the cryopreservation method on the third day. Furthermore, patients with more than six good-quality embryos on the third day will be counseled for extended blastocyst and culture transfer.

In addition, a failed-fresh cycle will be defined as a negative *β*-hCG test, fourteen days after fresh embryo transfer. Participants will then be randomized to immediate *versus* delayed groups by specially assigned nurses on the material day. They will also be advised on whether to wait for one or more menstrual cycles after the failed embryo transfer, before the next visit. Moreover, all participants will receive the same treatment before and after the subsequent visit, and there will be no other relevant interventions or concomitant care during the clinical trial. The nurses will inform patients before enrollment that once they are assigned to a group, they will not be able to change unless an unexpected event necessitates the cancellation of embryo transfer.

Furthermore, the participants’ stress and anxiety levels will be measured using a standard questionnaire before randomization and at the time of commencing FETs. In addition, the Chinese State-Trait Anxiety Inventory will be used to measure the patient’s anxiety levels (26).

On the other hand, Hormone Replacement Treatment (HRT) will be used for endometrial preparation. In addition, treatment with Estradiol valerate will commence on the third day of the menstrual cycle at a dose of 4–6 mg/day for 10–12 days. Moreover, vaginal progesterone (8% Crinone, Merck-Serono, Switzerland) will be administered at a dose of 90 mg per day, after the endometrial layer reaches a thickness of 8 mm as revealed by pelvic ultrasound scanning. FET for day three embryos will also be scheduled on the fourth day after commencing treatment with vaginal progesterone. Furthermore, at least one embryo with the best morphology, will be transferred using a soft embryo-transfer catheter with the guidance of ultrasound. Finally, the serum *β*-hCG levels will be checked fourteen days after FET.

### Data Collection and Follow-Up

A transvaginal ultrasound will be performed after two weeks if the serum *β*-hCG levels are positive to confirm fetal viability and identify intrauterine pregnancies. Participants will also be referred for antenatal care after twelve weeks. Additionally, the written consent and delivery data will be confirmed from patients at the time of the study. Moreover, participants will receive information about their pregnancy outcomes through a telephone call and or *via* a text message. After the retrieval of information by participants, pregnancy outcomes including the number of babies delivered, miscarriages, and birth weights will be recorded.

### Measurement of Outcomes

#### Primary Outcome

The primary outcome will be ongoing pregnancy, *i.e.* more than 12 gestational weeks of an intrauterine living fetus verified by pelvic ultrasonography.

#### Secondary Outcomes

Positive pregnancy, *i.e.* serum *β*-hCG level ≥10 mIU/ml.
Embryo implantation rate: the number of intrauterine gestational sacs observed divided by the number of embryos transferred.
Clinical pregnancy, defined as an intrauterine gestational sac with fetal heartbeat detected by transvaginal ultrasonography after 6 weeks of gestation.
Ectopic pregnancy, defined as a pregnancy in which implantation takes place outside the uterine cavity.
Miscarriage, defined as clinically recognized spontaneous loss of pregnancy before the completion of twenty gestational weeks.
Live birth, defined as the birth of at least one child with breath and heartbeat, irrespective of the duration of gestation.
Low birth weight, defined as <2,500 g of a newborn’s weight.

### Data Entry and Quality Control

Embryological laboratory data and treatment-related information including Controlled Ovarian Hyperstimulation (COH) and baseline characteristics will be collected on the day of embryo freezing. On the other hand, data on the FET cycle will be collected on the day of frozen embryo transfer. According to the study protocol, participants’ follow-up data will include that from enrollment and one year onwards. Moreover, patients’ information forms will be retrieved for data entry. Investigators will also be required to ascertain the data accuracy levels (first and second levels), as both levels will include data validation and monitoring, which will be conducted regularly throughout the study by the Data Monitoring Committee (DMC). Data will also be backed up to another computer within the same physical location as the server, on a daily basis.

### Statistical Analysis and Sample Size Calculations

#### Estimation of Sample Size

The PASS software version 11.0 (NCSS, LLC. Kaysville, Utah, USA.) will be used to calculate the sample sizes for both groups. Notably, the ongoing pregnancy rate per FET was about 30% based on data from our reproductive center, at the time of the trial design. The calculation revealed that 329 women were to be included in each trial group to provide an 80% power to detect a minimally important absolute difference of 10% points between the immediate and delayed groups for the primary outcome of ongoing pregnancy (40 *vs* 30%) at a two­sided *α* level of 0.05. However, the trial plans to include 732 women, with 366 participants in each arm in order to account for an expected 10% loss to follow-up.

#### Data Analysis

The intention-to-treat analysis will be used to examine differences in the ongoing pregnancy rate and other secondary outcomes in the two treatment arms during primary analysis. In addition, multiple imputations will be used to process missing values in the data. The demographic characteristics of the two groups will also be compared. Notably, categorical variables will be compared using χ2 analysis while the Student’s t-test will be used to compare quantitative variables. Moreover, multivariable logistic regression will be used to adjust for potentially confounding factors and results, if the randomization fails to achieve two balanced groups. Such factors will include the COH protocol, number of good-quality embryos, female age at oocyte-retrieval day, number of transferred embryos, retrieved oocytes, and quality of the transferred embryos. All statistical analyses will be performed using the SPSS version 26.0 software and a p-value <0.05 will be considered to be statistically significant.

### The Public and Patients Involvement

Participants’ who failed the fresh ET were the first to pose the question on the optimal timing for FET after stimulated IVF cycles. However, participants will neither be involved in designing nor conducting the study. Additionally, attending physicians will be tasked with disseminating the results to the respective participants.

## Dissemination and Ethics

HRT cycles are the standard procedure in IVF centers. In addition, there is currently no consensus with regard to the time interval between a failed-fresh ET and the subsequent FET. Therefore, there are no standardized predefined criteria for the premature termination of the study.

Women who agree to participate in the study will sign a consent form (see additional online file) after extensive counseling. They however will be at liberty to withdraw from the study at any given time, without prior notice.

Additionally, data will be electronically entered, stored, and locked in personalized computer files only accessible to the research staff and investigators involved in the study. Moreover, original study forms will be stored and maintained at the study site for a period of three years after completion of the research. The investigators will also allow trial-related Institutional Review Board/Independent Ethics Committee (IRB/IEC) reviews, audits, and monitoring of the data sources as well as the relevant documents. Moreover, participants will be required to contact their physician with regard to questions pertaining the study.

The primary objectives of the Data and Safety Monitoring Committee (DSMC) will be to; (i) ensure the safety of participants (ii) ensure the integrity of data (iii) interpret and review the generated data. The committee will consist of two independent researchers with experience in assisted reproductive medicine.

Moreover, an audit trail will be designed to preserve the integrity of the trial and will serve as an additional security measure. Herein, tracked changes will be implemented for the computer-generated and time-stamped audit trails in an electronic source document format. In addition, internal safeguards will be built into the computerized system.

The IEC/IRB approved the amended protocol of the study. Before implementation, safety and data were approved by the ethics committee.

The results of the study will be disseminated for publication through peer review.

## Discussion

In daily clinical practice, deciding on when to perform FET after a failed oocyte retrieval-ET procedure is often a dilemma. In addition, most of the patients prefer to continue treatment by postponing the transfer of their remaining cryopreserved embryos.

In a small retrospective cohort study (n = 129), Volodarsky-Perel et al. found that postponing FET by at least one menstrual cycle after a failed fresh ET was beneficial for pregnancy outcomes compared to immediate FET. This was observed if a preceding long GnRH-agonist protocol for COS was used and if an artificial preparation was indicated for FET (22). However, these results were contrary to those recently reported by a large retrospective study (n = 1,183) which revealed a similar clinical pregnancy rate in first FET cycles that were performed immediately (≤22 days) or later on (>22 days) after oocyte retrieval from the preceding failed fresh ET (21). The study concluded that deferring FET may unnecessarily prolong the time to pregnancy. Nevertheless, the results were drawn from cycles that followed failed Fresh ET using the ovarian stimulation-GnRH-ant protocol. All in all, the contradictory results from the two studies may have been due to the different protocols used for ovarian stimulation, *i.e.* GnRH analog in the failed fresh ET and in the subsequent first FET cycle.

The purpose of this randomized controlled trial is not necessarily to confirm the superiority or inferiority of immediate FET after the failure of fresh ET. On the contrary, it aims to assure both clinicians and patients that although fresh ET fails, immediate FET has no adverse effects on the eventual pregnancy outcomes, compared to delayed FET. As such, it may be beneficial in alleviating psychological pressure on patients and shorten the Time to Pregnancy (TTP) and is therefore worth trying.

## Trial Status and Peer Review

This trial is at Version 1.7, 28/5/2020 (ChiCTR). The study was designed on the 10^th^ of May, 2020, and the first participants were randomized on the 15^th^ of October, 2020.

The actual study start date was 1 June 2020, and it is anticipated to end on 31 May 2021. In addition, recruitment began on 15 June 2020, and it was anticipated to end on 15 October 2020. At the time of the manuscript preparation, the study will have recruited 310 infertile women and recruitment currently is ongoing. This study is externally peer-reviewed and does not include commission.

## Ethics Statement

The study was approved by the Ethics Committee of Assisted Reproductive Medicine at the Affiliated Hospital of Shandong University of traditional Chinese medicine (SDTCM/E-2020.2.01). Written informed consent was obtained from all participants before the study. The results of this trial will be disseminated in a peer-reviewed journal. Trial registration number: ChiCTR2000033313.

## Author Contributions

Study design: JS and ZL. Analysis and interpretation of data: JS and FD. Collection and management of data: YXW and MZ. Initial draft and review of the study: ZL, YHW and JS. Ethical approval: Sorted by ZL and MZ. Study coordination and recruitment of subjects: MS and YHW. All authors contributed to the article and approved the submitted version.

## Funding

The study is supported by the Natural Science Foundation of Shandong Province (ZR2016HP44). The funder was not involved in the study design, collection, analysis, interpretation of data, the writing of this article or the decision to submit it for publication.​​​​​​

## Conflict of Interest

The authors declare that the research was conducted in the absence of any commercial or financial relationships that could be construed as a potential conflict of interest.
